# Differentiating active from Inactive Sacroiliitis in ankylosing spondylitis by combination of DWI and Magnetization Transfer Imaging

**DOI:** 10.12669/pjms.39.2.6094

**Published:** 2023

**Authors:** Qiuping Ning, Tiebing Fan, Hua Ren, Huiyi Ye, Wensheng Wang

**Affiliations:** 1 Qiuping Ning, Medical School of Chinese PLA, Beijing, 100853, China; Department of Radiology, The First Medical Centre, Chinese PLA General Hospital, Beijing, 100853, China, Department of Radiology, China Academy of Chinese Medical Sciences Xiyuan Hospital, Beijing, 100091, China; 2 Tiebing Fan, Postdoctoral Management Office, Chinese Academy of Chinese Medical Sciences, Beijing, 100853, China; 3 Hua Ren, Department of Radiology, China Academy of Chinese Medical Sciences Xiyuan Hospital, Beijing, 100091, China; 4 Huiyi Ye, Department of Radiology, The First Medical Centre, Chinese PLA General Hospital, Beijing, 100853, China; 5 Wensheng Wang, Department of Radiology, China Academy of Chinese Medical Sciences Xiyuan Hospital, Beijing, 100091, China

**Keywords:** Ankylosing Spondylitis, Magnetic Resonance Imaging, Diffusion Magnetic Resonance Imaging, Magnetization Transfer Contrast Imaging

## Abstract

**Objectives:**

To evaluate lesions of sacroiliac joint (SIJ) by combination of diffusion-weighted imaging (DWI) and magnetization transfer (MT).

**Methods:**

A retrospective study was used in this study. Forty-nine ankylosing spondylitis (AS) patients admitted to The China Academy of Chinese Medical Sciences Xiyuan Hospital from May 2020 to October 2020 were collected into active and inactive groups. Twenty-two healthy volunteers were recruited. Apparent diffusion coefficient (ADC) values for bone marrow edema (BME), sclerosis area, fat deposit area, and normal-appearing bone marrow (NABM) (both patients and healthy volunteers) and the magnetization transfer (MT) rate of the cartilage (MTRc) were analyzed in the groups. The above five parameters (ADC (NABM), ADC (BME) and ADC (fat deposit) and MTRc) between the active group and the inactive group were compared. The effectiveness of each parameter in diagnosing sacroiliac arthritis of ankylosing spondylitis were analyzed, and the predictive value of the parameters was compared.

**Result:**

ADC(BME), ADC(NABM) and MTRc showed statistically significant differences between the active and inactive groups (*P* <0.05). ADC (BME) and ADC (NABM) could predict the activity of AS sacroiliac arthritis (*P* <0.01). ADC (NABM) and MTRc were significantly different between healthy volunteers and the active group (*P* <0.01). The areas under the ROC curve (AUCs) of ADC (BME)_ADC(NABM), ADC(NABM)_MTR, and ADC(BME)_MTRc were 0.885 (cut-off value=0.69), 0.849 (cut-off value=0.56) and 0.864 (cut-off value=0.60), respectively. The predictive ability of the combined index ADC (BME)_MTRc and ADC(NABM)_MTRc was increased.

**Conclusion:**

The ability to diagnose and predict AS might be improved by the combination of diffusion-weighted imaging (DWI) and magnetization transfer (MT).

## INTRODUCTION

The activity detection of Ankylosing spondylitis (AS) is necessary for the treatment and follow-up. Diffusion-weighted imaging (DWI) is sensitive to bone marrow edema (BME) under the joint surface of AS^1^ and is effective in the assessment of sacroiliac arthritis in AS patients and the treatment effect during follow-up.^2^,^3^ DWI may provide superior specificity to MRI in detecting and monitoring the activity of sacroiliac arthritis.^4^ Therefore, MRI is an effective robust method for the detection of AS activity.

Magnetization transfer (MT) is a phenomenon observed in MRI and is a generally accepted tool in neurology.^5^ MT can be used to examine articular cartilage. Studies have reported that the sensitivity of magnetic transmittance in spinal arthritis patients with negative sacroiliac arthritis was approximately 83.3%, but the specificity was low.^6^ In this study, DWI and MT were combined to identify active AS disease by SIJ lesions to detect sacroiliac arthritis at an early stage.

## METHODS

This was a retrospective diagnostic study. Patients with AS admitted to The China Academy of Chinese Medical Sciences Xiyuan Hospital from May 2020 to October 2020 were included into active and inactive groups. The study was approved by the Institutional Ethics Committee of China Academy of Chinese Medical Sciences Xiyuan Hospital on (No.: 2019XLA041-2, date:23-07-2019).

### Inclusion criteria:

1) Meeting the modified Ankylosing Spondylitis International Society (ASAS) diagnostic criteria for AS,^7^
2) The detection of BME.

### Exclusion criteria:

1) Sacroiliac joint lesions such as tumors, metastasis, tuberculosis, etc.,
2) Thyroid disease, hyperparathyroidism, and bone metabolism-related diseases,
3) Taking drugs that affect bone metabolism,
4) Poor signal-to-noise ratio and image quality for analysis,
5) MRI-related contraindications such as metal implants or claustrophobia. Healthy volunteers were also recruited as a control group.

### Clinical diagnosis:

The gold standard of diagnosis in this study is clinical diagnosis. According to the Ankylosing Spondylitis International Society (ASAS) diagnostic criteria, patients with an average Bath Ankylosing Spondylitis Disease Activity Index (BASDAI) score ≥6 or a BASDAI score between 4.0-6.0, an ESR ≥20mm/h or a CRP level ≥6.0mg/L were included in the active group.^8^

### Laboratory Assessments:

Routine venous blood collection was performed. Hematological indicators included ESR, CRP, and HLA-B27.

According to the above grouping criteria, there were 32 patients in the active group, and 17 patients in the inactive group. The general data of the two groups (including gender composition ratio, average age, average age of onset and average course of disease) showed no statistically significant difference and were comparable, as shown in Table-I.

**Table-I:** Clinical Characteristics of the AS Patients (Active and Inactive Groups) Compared by Independent Samples T Tests.

	Active (n=32)	Inactive (n=17)	P
Age (years)	32.9±9.2 (22-51)	35.1±9.5 (22-52)	0.624
Males	19 (73.1%)	8 (61.5%)	0.486
Weight (kg)	64.6±11.0	67.1±9.9	0.500
Related comorbidities			
Uveitis	9 (34.6%)	8 (61.5%)	
Urinary calculi	9 (34.6%)	5 (38.5%)	
Aplastic anemia	1 (3.8%)	0	
SAPHO	1 (3.8%)	0	
Family history	5 (19.2%)	2 (15.4%)	
Onset age (years)	20.7±5.3	20.2±2.5	0.386
Symptom duration (years)	9.8±5.9	10.8±8.8	0.953
HLA-B27 positive rate	69.2%	30.8%	<0.001
BASDAI	5	3	<0.001
ESR (mm/h)	11	5.3	0.581
CRP (mg/L)	23	5.7	0.001

BASDAI: Bath Ankylosing Spondylitis Disease Activity Index, ESR: erythrocyte sedimentation rate, CRP: C-reactive protein, SAPHO: synovitis-acne-pustulosis-hyperostosis-osteomyelitis syndrome.

*Test* MRI: MRI examinations were performed with a 3-T system, using an eight-channel body phase control matrix coil. Patients were placed in the supine position. The parameters were as follows:

a) oblique axial DW, TR/TE=2400/10.0, echo train length=128, flip angle=90º, FOV=38×38 cm, matrix=128×128, band width=250 kHz, thickness/gap=4.0/0.4, NEX=2, time=77s, b value=0 s/mm^2^ and 800 s/mm^2^, spin-echo echo-planar imaging (SE-EPI), fat suppression, two-dimensional (2D) collection, diffusion directions=3, orientation=ALL, and acceleration technique=ASSET (phase acceleration factor: 2), scan time=1 min 17s.

b) oblique-coronal MT contrast, spoiled gradient echo (SPGR) sequence, TR/TE=50.0/12.0, flip angle=10º, FOV=38×38 cm, matrix=256×224, band width=31.25 kHz, thickness/gap=2.0/0.0, NEX=1, time=137s. The MT pulse duration was 8ms. The MT frequency offset was 1200 Hz. The MT flip angle was 670. The MT pulse type was Fermi. Both series used 2D collection without adding fat suppression.

### Image post-processing and region of interest (ROI):

**Fig.1:**
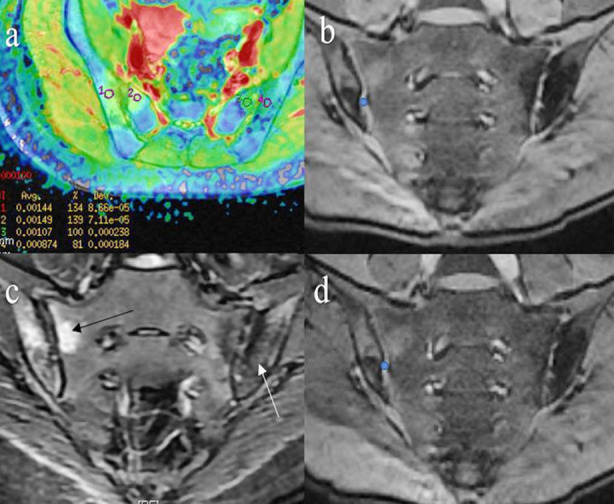
Patients in the acute group. A 32-year-old female AS patient with right lumbosacral pain and morning stiffness, onset age 26 years, HLA-B27-positivity, and no family history of AS. Combined T2WI and DWI image. (a) and FS-T2WI image (b) showing articular sclerosis (white arrow) and BME (black arrow) of the sacroiliac joints in one patient. The location of the region of interest (ROI) is shown in (a). The ROI is selected in the sclerotic region from within 1 mm of the cortices of the bilateral ilia and sacrum, areas of bone erosion, blood vessels, and cysts were avoided. The mean values after multiple measurements were used for the analysis. For magnetic transfer (MT) images, the ROI was selected within the inferior ventral joint spaces of the bilateral sacroiliac joints (c: pre-saturation pulse (M0), (d): after the pre-saturation pulse (Ms) (blue circles)).

Two radiologists with rich experience in the diagnosis of musculoskeletal system imaging read the films. a) **DWI:** The images were transferred to a GE Advantage Workstation 4.6 to generate ADC images using the MADC calculation. The images with b=800 s/mm^2^ and T2WI images were registered (Fig.1a). The ROI was placed at the largest level of the lesion on the image after registration. The ROI area was about 15-30 mm^2^. The ADC values of the edema area, sclerosis area (Fig.1b), fat deposit area, and normal-appearing bone marrow were recorded as ADC(BME), ADC (sclerosis), ADC (fat deposit) and ADC(NABM), respectively.

b) **MT:** The viewer function in the GE Advantage Workstation 4.6 was used to analyze the image before applying the pre-saturation pulse (M0) (Fig.1c) and the image after applying the pre-saturation pulse (Ms) (Fig.1d). The ROI was placed in the same place as the ventral articular cartilage in the anterior lower part of the bilateral SIJ. The area was 1-3 mm^2^. The signal intensity of the articular cartilage of the SIJ at the same layer was measured before and after applying the pre-saturation pulse. According to the MTR formula, the magnetization transfer rate of the cartilage (MTRc) was calculated: MTR=(M0-Ms)/M0×100%.

### Statistical Analysis:

SPSS 22.0 and MedCalc15.2.2 were used for the statistical analyses. T test was used for measurement data. The ROC curve was used to compare the predictive values of the indicators between the two groups. Logistic regression was used to combine the indicators with predictive value and the Z test was used to compare the area under the curve (AUC) differences of the combined indicators. *P*<0.05 was considered to indicate statistical significance.

## RESULTS

The HLA-B27 positive rate, BASDAI, and CRP levels were significantly different between the active and inactive groups (*P*<0.001), whereas age, sex, onset age, disease duration, and ESR did not significantly differ between the groups (*P*>0.05) (Table-I).

The results showed that the ADC(BME), ADC(NABM), and MTRc values displayed high interobserver agreement between the two radiologists, while poor interobserver agreement was observed for ADC (sclerosis) and ADC (fat deposit) in the active group (Table-II).

**Table-II:** Mean ADC and MTRc Values of the Active and Inactive Sacroiliitis Patients and Healthy Volunteers.

	Active Sacroiliitis mean±SD (range) (n=32)	Inactive Sacroiliitis mean±SD (range) (n=17)	Healthy volunteers mean±SD (range) (n=22)	P
ADC(NABM) (×10^-3^ mm^2^/s)	0.35±0.12(0.15-0.58)	0.50±0.07(0.36-0.59)	0.56±0.17(0.36-0.97)	0.000^a^ <0.001^b^ 0.576^c^
ADC(BME) (×10^-3^ mm^2^/s)	0.83±0.26(0.40-1.19)	0.61±0.10(0.44-0.79)	/	0.016
ADC(sclerosis)	0.41±0.36(0.71-0.91)	0.53±0.10(0.34-0.74)	/	0.120
ADC(fat deposit)	0.45±0.26(0.36-0.89)	0.55±0.05(0.39-0.76)	/	0.132
MTRc	0.30±0.09(0.10-0.41)	0.38±0.13(0.17-0.52)	0.44±0.13(0.20-0.78)	0.025^a^ <0.001^b^ 0.118^c^

a: comparison between active and inactive groups, b: comparison between active and healthy volunteer groups, c: comparison between inactive and healthy volunteer groups. ADC: apparent diffusion coefficient, NABM: normal-appearing bone marrow, BME: bone marrow edema, MTRc: magnetic transfer rate of cartilage.

BME was detected in both the active group and the inactive group, with 75 BME sites in the active group and 25 in the inactive group. In the active group, 12 patients had no sclerosis lesions, and a total of 16 patients had 88 sclerosis sites (Fig.1b). Good agreement was found between the two radiologists for BME, while poor agreement was observed for sclerosis and cartilage lesions (Table-II).

The values for ADC (BME), ADC (NABM) and MTRc were statistically significant (*P*<0.05). There was no significant difference between the two groups for the ADC (sclerosis) and ADC (fat deposit) values. The values for ADC (NABM) and MTRc were significantly different between the healthy volunteers and the active group (*P*<0.001) (Table-II).

**Fig.2:**
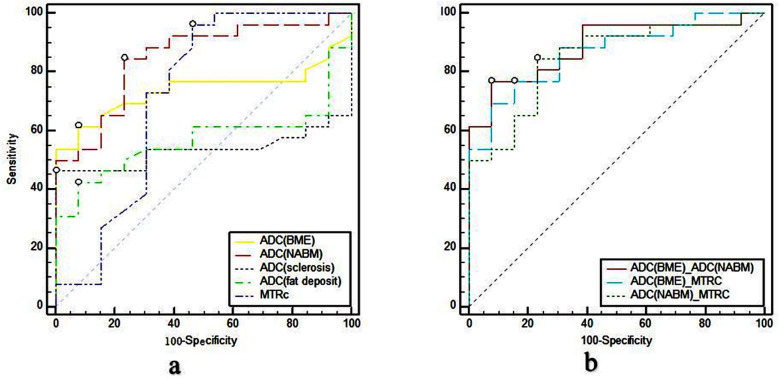
(a-b) Respectively indicate the ROC curves of MTRc, ADC (BME), ADC (sclerosis), ADC (fat deposit), and ADC (NABM) for predicting the activity values of sacroiliitis. Order of AUCs: ADC (NABM)>ADC (BME)>MTRc>ADC (fat deposit)>ADC (sclerosis). (c) Shows the ROC curves of ADC(BME)_ADC(NABM), ADC(NABM)_MTRc, and ADC(BME)_MTRc for the prediction of active sacroiliitis in ankylosing spondylitis. The AUC of ADC (BME)_ADC(NABM) was the greatest (AUC=0.885). The order of the three indices is ADC (BME)_ ADC(NABM)>ADC(BME)_MTRc>ADC(NABM)_MTRc. BME: bone marrow edema, NABM: normal-appearing bone marrow, MTRc: magnetization transfer rate of cartilage.

The value of ADC (BME), ADC (sclerosis), ADC (fat deposit), ADC(NABM) and MTRc for diagnosing AS activity. Table-II. The results show that MTRc cannot predict AS activity alone (Fig.2a). ADC (BME) and ADC (NABM) can predict AS sacroiliac arthritis activity (Fig.2a). The ROC curve analysis of the two groups is shown in Table-III.

**Table-III:** DWI and MT, and the Value of Laboratory Indicators Alone and Combined for Predicting the Activity of Ankylosing Spondylitis and Sacroiliitis.

	AUC	SE	95% CI	P	Youden index (sensitivity, specificity)
ADC(BME)	0.738	0.081	0.589-0.896	0.003	0.54 (61.5, 92.3)
ADC(NABM)	0.849	0.064	0.723-0.975	<0.0001	0.62 (84.6, 76.9)
MTRc	0.719	0.107	0.509-0.929	0.041	0.50 (96.2, 53.9)
BASDAI	0.883	0.056	0.774-0.993	<0.0001	0.65 (88.5, 76.9)
ESR	0.732	0.087	0.562-0.903	0.008	0.42 (88.5, 53.9)
CRP	0.784	0.098	0.593-0.975	0.004	0.65 (96.2, 69.2)
ADC(BME)_ ADC(NABM)	0.885	0.053	0.781-0.989	<0.0001	0.69 (76.9, 92.3)
ADC(NABM)_MTRc	0.849	0.0635	0.725-0.974	<0.0001	0.62 (84.6, 76.9)
ADC(BME)_MTRc	0.864	0.0575	0.751-0.977	<0.0001	0.62 (76.9, 84.6)

AUC: area under the receiver operating characteristic curve, SE: standard error, BME: bone marrow edema, NABM: normal-appearing bone marrow, MTRc: magnetization transfer rate of cartilage.

The AUC of the combination index were ADC (BME) MTRc=0.864 and ADC (NABM) MTR=0.849. The area difference was 0.0148 (95% confidential interval (CI): -0.144-0.174, *Z* value=0.182), the difference was not statistically significant (P=0.8555). The diagnostic efficiency of ADC (BME) MTRc was better than that of ADC (BME) and MTRc alone, and the AUC of ADC (NABM) MTRc was equal to that of MTRc (Fig.2b).

## DISCUSSION

The use of contrast-enhanced MRI is limited in patients with AS because of the frequent association of AS with glomerulopathies.^9^ MT and DWI technologies can be used to quantitatively determine AS activity without a contrast agent. Ostrowska et al.^10^ Observed that BME represents an important indicator of bone destruction and could reflect inflammatory activity. BME can result in the following: a.) an increase in the proportion of water molecules in the extracellular space, b.) an increase in the blood transported by capillaries to the bone marrow cavity, and c.) an increase in the diffusion movement of water molecules.^11^ Our study was consistent with a previous study in that the ADC values of lesions were higher than those of the unaffected normal bone marrow (ADC(NABM)) in AS patients.^12^ The ADC(BME) value of the active group was higher than that of the inactive group. In our study, the ADC(NABM) value of the healthy volunteer group was higher than that in active group and inactive group, and ADC(NABM) showed especially lower values in the active sacroiliitis group, which was inconsistent with the studies of Gezmis et al.^13^ The reason for this finding was that the ADC(NABM) value of the active group was influenced by erosion and periarticular fat deposits.

The ICC of ADC (sclerosis) was relatively low, probably because sclerosis often coexists with BME and the two entities can easily be mistaken.^14^ In clinical practice, when osteosclerosis and BME occur simultaneously, the specificity of active inflammation observed by MRI is lower than that of BME alone.^15^ Accuracy could be improved by using multiple measurements and ROI reduction. The ADC (fat deposit) value in the inactive group was higher than that in the active group in our study. Fat depositions and sclerosis are considered chronic structural changes resulting from sacroiliitis and are not findings of active processes.^16^ Thus, ADC (fat deposit) and ADC(sclerosis) cannot be used for the detection of sacroiliac arthritis activity. Co-registration images (T2WI+DWI) were used to place the ROIs and for the measurements. Co-registration massively influences the independence of DWI data, making it more dependent on observer subjectivity and observations made on other sequences. In this study, the b value=800×10^-3^ mm^2^/s was used, and the reproducibility of ADC (NABM), ADC (BME) and ADC (fat deposit) was good (ICC>0.75), all lesions were clearly displayed and adequate for diagnosis, except for ADC (sclerosis). Studies have shown that the ability of ADC values to detect activity is significantly higher than the quantitative parameters generated by all biexponential models. And ADC values of water are much greater than ADC values of fat.^17^ In this study, we use monoexponential models to estimate ADC values, and used fat suppression.

MT can be used in quantitative analyses of the basic macromolecular dynamics and chemistry of tissues. As inflammation progresses, MTR could even reduce to close to zero.^18^ In this study, the MTRc in the active group was significantly lower than that in the inactive group, which is consistent with previous conclusions.^6^ MTRc has better sensitivity than ADC (BME) and ADC(NABM) but lower specificity. DWI and STIR are sensitive for detecting bone marrow edema, and the activity of the lesions can be preliminarily judged based on the high signal of the above sequence. We combined marrow edema and cartilage destruction to improve the diagnostic specificity of active AS in clinical practice. After combination, the AUC of ADC(BME)_MTRc was greater than that of ADC(NABM)_MTRc, and we confirmed that BME is not specific to active AS. MT took 274 s in our research, which could prolong the exam procedure and possibly lead to movement artifacts and discomfort. MT should be abandoned if it caused discomfort to the patients. In recent years, pathophysiological technology using glycosaminoglycan has been developed, such as delayed gadolinium-enhanced MRI of cartilage (dGEMRIC)^19^ and glycosaminoglycan chemical exchange saturation transfer (gagCEST)^20^, but technical limitations hinder the popularization and clinical application of these emerging technologies.

### Limitations

First, this was a retrospective study with a relatively small sample size. Second, partial volume may affect ADC measurements in small lesions. Third, some lesions presented with predominantly cystic changes and necrosis, which may affect the ADC values or signal intensity measurements. Finally, for ethical reasons, no pathological results were available for reference.

## CONCLUSION

DWI and MT have certain value in the detection of AS sacroiliitis activity. ADC(NABM) and ADC(BME) can be used to identify the activity of sacroiliac arthritis in AS. After combination, the diagnostic efficacy of ADC(BME)_MTRc and ADC(NABM)_MTRc was improved.

### Authors’ Contributions:

**QN &** **HY:** Designed this study, prepared this manuscript, are responsible and accountable for the accuracy and integrity of the work.

**TF:** Collected and analyzed clinical data.

**HR & WW:** Data analysis, significantly revised this manuscript.
